# DNA damage is overcome by TRIP13 overexpression during cisplatin nephrotoxicity

**DOI:** 10.1172/jci.insight.139092

**Published:** 2021-11-22

**Authors:** Taketsugu Hama, Prashanth K.B. Nagesh, Pallabita Chowdhury, Bob M. Moore, Murali M. Yallapu, Kevin R. Regner, Frank Park

**Affiliations:** 1 Department of Pharmaceutical Sciences, College of Pharmacy, University of Tennessee Health Science Center, Memphis, Tennessee, USA.; 2 Department of Immunology and Microbiology, School of Medicine, University of Texas Rio Grande Valley, McAllen, Texas, USA.; 3 Division of Nephrology, Department of Medicine, Medical College of Wisconsin, Milwaukee, Wisconsin, USA.

**Keywords:** Nephrology, DNA repair, Mouse models

## Abstract

Cisplatin is a commonly used chemotherapeutic agent to treat a wide array of cancers that is frequently associated with toxic injury to the kidney due to oxidative DNA damage and perturbations in cell cycle progression leading to cell death. In this study, we investigated whether thyroid receptor interacting protein 13 (TRIP13) plays a central role in the protection of the tubular epithelia following cisplatin treatment by circumventing DNA damage. Following cisplatin treatment, double-stranded DNA repair pathways were inhibited using selective blockers to proteins involved in either homologous recombination or non-homologous end joining. This led to increased blood markers of acute kidney injury (AKI) (creatinine and neutrophil gelatinase–associated lipocalin), tubular damage, activation of DNA damage marker (γ-H2AX), elevated appearance of G2/M blockade (phosphorylated histone H3 Ser10 and cyclin B1), and apoptosis (cleaved caspase-3). Conditional proximal tubule–expressing *Trip13* mice were observed to be virtually protected from the cisplatin nephrotoxicity by restoring most of the pathological phenotypes back toward normal conditions. Our findings suggest that TRIP13 could circumvent DNA damage in the proximal tubules during cisplatin injury and that TRIP13 may constitute a new therapeutic target in protecting the kidney from nephrotoxicants and reduce outcomes leading to AKI.

## Graphical abstract

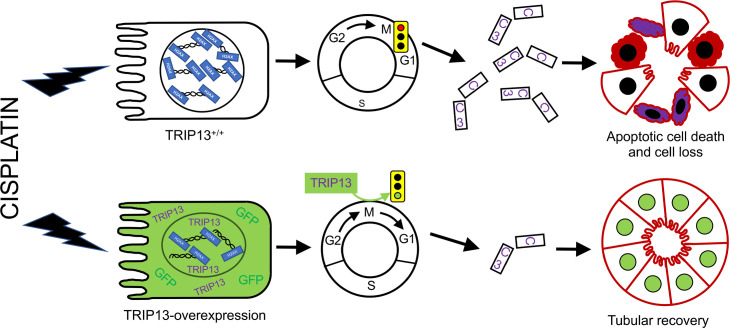

## Introduction

Acute kidney injury (AKI) is a major complication in nearly one-third of all cancer patients treated with cisplatin, a common chemotherapeutic drug used for a variety of human cancers (1–3). The development of nephrotoxicity is attributed to the rapid accumulation of cisplatin by active transport into renal tubular epithelial cells, particularly in the S3 segment of the proximal tubules (4, 5). The accumulation of cisplatin causes DNA cross-linking and, in some cases, more lethal physical breaks of the DNA (1, 6–10). These types of damage to the genomic DNA play a critical role in the phenotypic changes exerted on tubular epithelial cells and any subsequent loss of renal function (11, 12). To preserve normal function of the cells, checkpoint systems have evolved to maintain genome stability by activating surveillance systems to ensure that damaged DNA from previous cell cycle phases are not transferred into mitosis. In the kidney, however, our understanding of these processes is largely undescribed, and in the case of AKI, the activation of certain DNA repair systems does not necessarily lead to a beneficial outcome to preserve or even restore renal function (13, 14).

Thyroid receptor interacting protein 13 (TRIP13) is an evolutionarily conserved family member of the AAA^+^ ATPase enzymes (15, 16) and acts as an adapter protein with pleiotropic roles in pathways relevant to meiotic/mitotic checkpoint control (17–22), DNA recombination (15, 16), and double-stranded DNA repair (23). Insufficient production of *Trip13* can increase cell cycle blockade at the G2/M phase (23) and inefficiently repair double-stranded DNA breaks (16, 24, 25), which leads to the activation of apoptotic cell death pathways in germline and somatic cells (16, 23–25). Moreover, TRIP13 directly activates DNA-dependent protein kinase catalytic subunit (DNA-PKcs), which is a primary enzyme involved in the non-homologous end joining (NHEJ) pathway of double-stranded DNA repair, to promote tumorigenesis (23). In the kidney, global *Trip13^Gt/Gt^* hypomorph mice exhibit prolonged tubular epithelial cell damage following bilateral ischemia/reperfusion injury, which is associated with increased DNA damage and activated apoptotic signaling (26). To date, however, the role of TRIP13 to prevent tubular damage in other forms of AKI, such as cisplatin nephrotoxicity, with respect to double-stranded breaks has yet to be studied.

In the present study, pharmacological inhibitors targeting double-stranded DNA repair pathways were tested to determine any interference in the normal recovery of damaged renal tubules due to cisplatin. To determine whether TRIP13 could modulate this process, a transgenic mouse model was generated to conditionally overexpress *Trip13* using the GGT1-Cre recombinase, since the main segment of cisplatin nephrotoxicity is the proximal tubules. The *Trip13* transgenic mice appeared normal with no evidence of any abnormal renal morphology or function. In the face of AKI following cisplatin administration, the *Trip13*-expressing kidneys were largely protected from injury with no apparent loss of renal function compared to their wild-type littermates. This study provides information about TRIP13 as a renal protectant from nephrotoxic agents and suggests that this may be a new therapeutic target for AKI by circumventing double-stranded DNA breaks during the recovery of the damaged renal tubules.

## Results

### Conditional Trip13 transgenic mouse model.

**Figure 1:**
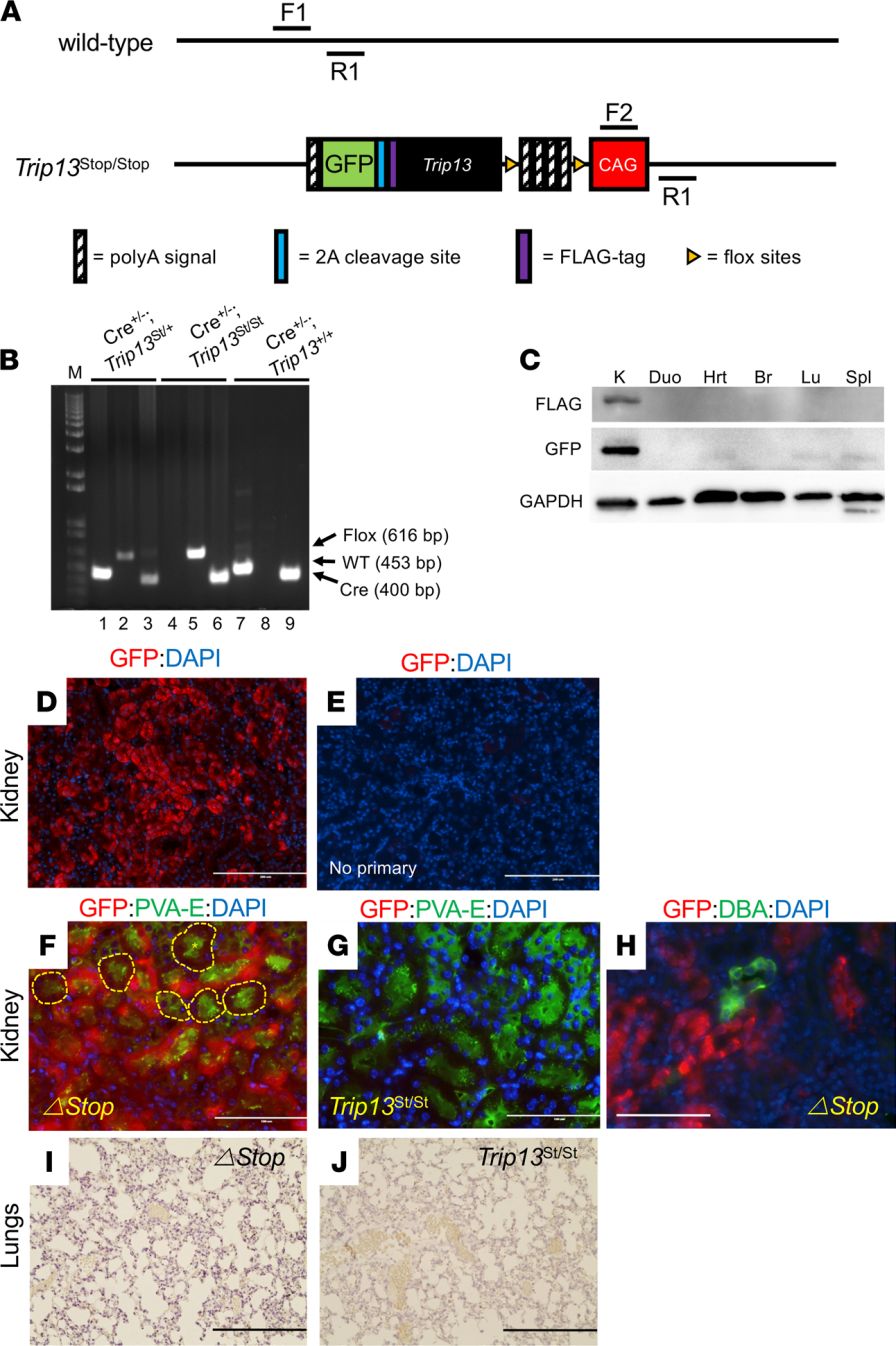
Genomic targeting construct of *Trip13* overexpression. (**A**) Targeting construct was designed for integration into the ROSA26 region, and the expression cassette was designed with the CAG promoter-floxed stop codon-Trip13 cDNA-T2A-EGFP-polyA. 2A, protease cleavage site (*Trip13* cDNA was FLAG tagged at the 3′ end); CAG, CMV-IE enhancer/chicken β-actin/rabbit β-globin. (**B**) Genomic DNA isolated from mouse pups was PCR analyzed using specific primers to differentiate wild-type, floxed stop, and Cre-containing mice. Wild-type (WT) = 453 bp; floxed stop (flox) = 616 bp; and Cre = 400 bp. M = 1 kb ladder; lanes 1, 4, and 7 = F1/R1 primers (wild-type); 2, 5, and 8 = F2/R1 primers (floxed Trip13); and 3, 6, and 9 = GGT1-Cre primers (Cre); *Trip13*^st/st^, *Trip13*^Stop/Stop^. (**C**) Western blot analysis of FLAG-tagged TRIP13 and GFP protein expression in harvested tissues from *Trip13*^ΔStop^ mice. GAPDH was shown as a loading control. K, kidney; Duo, duodenum; Hrt, heart; Br, brain; Lu, lungs; Spl, spleen. (**D**–**H**) Immunofluorescence was performed on FFPE *Trip13*^Stop/Stop^ and *Trip13*^ΔStop^ kidneys to detect GFP expression. GFP was detected by Alexa Fluor 555 fluorescence (red color) in (**G**) *Trip13*^Stop/Stop^ and (**D**, **F**, and **H**) *Trip13*^ΔStop^ kidneys. **E** shows a negative control kidney section incubated without primary GFP antibody (no primary). To determine proximal tubules (**F** and **G**) or collecting ducts (**H**), Alexa Fluor 488 (green) fluorescence was detected using PVA-E (brush border of proximal tubules) or DBA (collecting ducts). Nuclei were stained with DAPI (blue). Dashed lines in **F** indicate PVA-E–positive tubules with minimal to undetectable expression of GFP, showing mosaicism of Cre expression. (**I** and **J**) Immunohistochemical staining for GFP in cisplatin-treated *Trip13*^ΔStop^ and *Trip13*^Stop/Stop^ mouse lungs. All DAB-stained sections were counterstained with hematoxylin. Scale bar: 200 μm (**D**, **E**, **I**, and **J**), 100 μm (**F**–**H**). PVA-E, *Phaseolus vulgaris* erthyroagglutinin; DBA, *Dolicus biflorus* agglutinin.

A floxed stop codon strategy was employed to produce a conditional transgenic mouse model overexpressing *Trip13*. A DNA-targeting construct was designed and cloned as shown in Figure 1A. In our approach, multiple stop codons were cloned between flanking *loxP* sites, which were placed between the CAG promoter and the transgene cassette (*Trip13*-T2A-EGFP). This construct was injected for integration into the ROSA26 region into the zygotes, and litters were tested by Southern blot analysis for proper DNA banding following genomic DNA restriction digest. Founder heterozygote *Trip13*^Stop/+^ males and females were interbred to obtain homozygous *Trip13*^Stop/Stop^ mice. GGT-Cre mice were used to breed with the heterozygous *Trip13*^Stop/+^ mice to generate *GGT-Cre^+/–^* *Trip13*^Stop/+^ mice. Subsequently, the *GGT-Cre^+/–^* *Trip13*^Stop/+^ mice were bred with the homozygous *Trip13*^Stop/Stop^ mice to produce littermates of homozygous *Trip13*^Stop/Stop^ and *GGT-Cre^+/–^* *Trip13*^Stop/Stop^ (*Trip13*^ΔStop^) mice for our study. All mice were genotyped by PCR using specific primers shown in Table 1 and a representative ethidium bromide–stained agarose gel showing different genotyped mouse pups (Figure 1B).

Phenotyping of the mice was performed by detecting FLAG-tagged TRIP13 and GFP by Western blot analysis using protein lysates from harvested organs from female *Trip13*^ΔStop^ mice (Figure 1C). FLAG and GFP protein were clearly detected in the kidney lysates from *Trip13*^ΔStop^ mice with undetectable expression in the other organs tested, including duodenum, heart, lung, brain, and spleen. To determine the cell type(s) expressing GFP in the kidneys, we performed immunohistology using 2 different detection methods. Fluorescent antibodies were tested on *Trip13*^ΔStop^ mouse kidneys to determine whether GFP (shown in red) (Figure 1, D, F, and H) would colocalize with either proximal tubule–specific lectin PVA-E (green brush border) (Figure 1, F and G) or collecting duct marker, DBA (Figure 1H). In the *Trip13*^Stop/Stop^ mouse kidneys (Figure 1G), there was minimal Alexa Fluor 555 intensity, demonstrating the lack of GFP expression without the presence of Cre recombinase (Figure 1H). Similarly, minimal detection of GFP signal was detected in *Trip13*^ΔStop^ mouse kidneys in the absence of the primary antibody (negative control image in Figure 1E). Immunostaining was absent in the lungs from both *Trip13*^ΔStop^ (Figure 1I) and *Trip13*^Stop/Stop^ (Figure 1J) mice, consistent with the lack of GFP staining by Western blot analysis (Figure 1C). Similar results were obtained using DAB staining (data not shown), showing the segment-specific expression of TRIP13 and GFP in our generated mouse model.

### Overexpression of Trip13 in proximal tubules prevents the loss of renal function following cisplatin administration.

**Figure 2:**
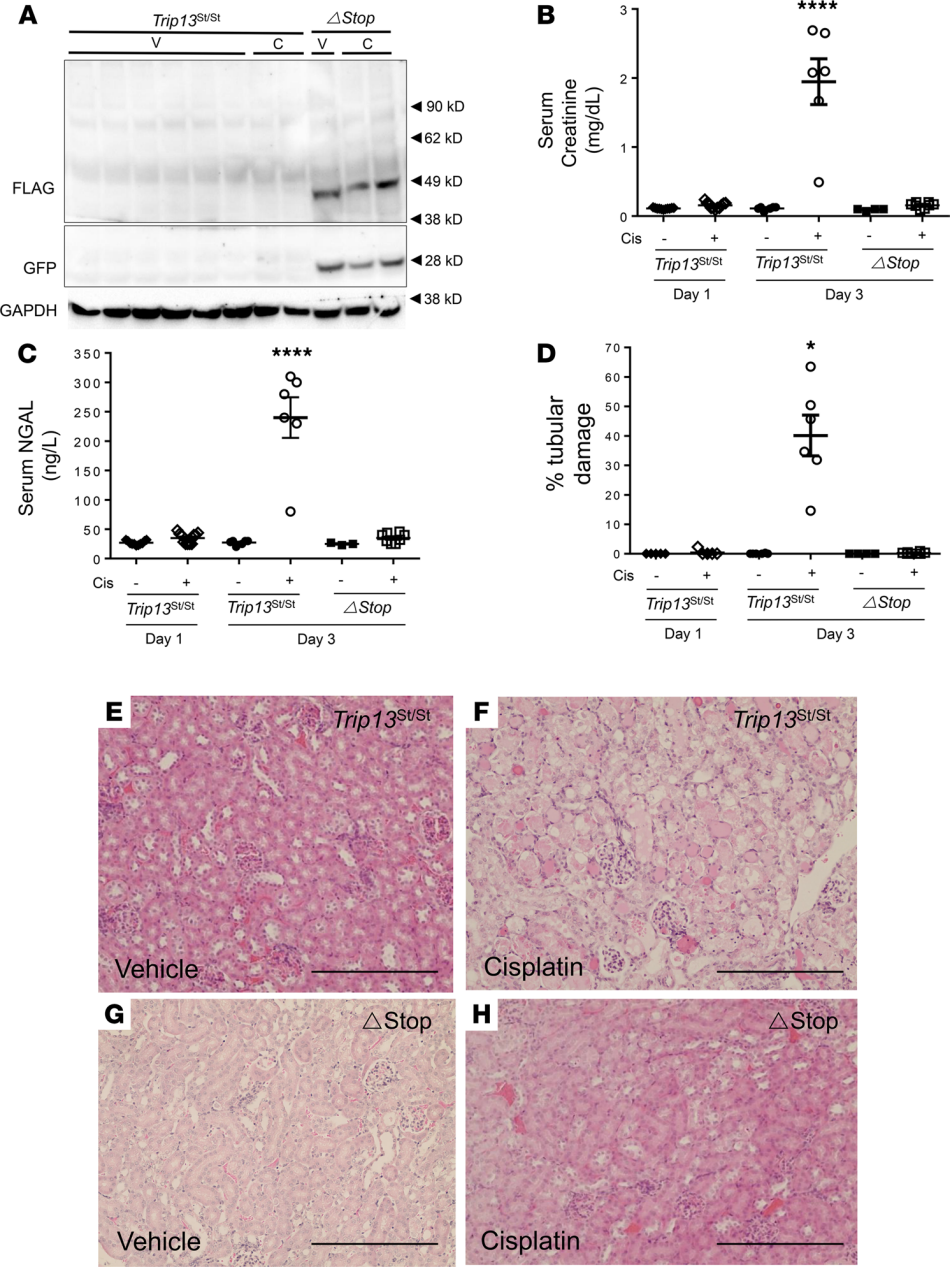
*Trip13* overexpression in the proximal tubules provides protection from renal tubular epithelial cell damage following cisplatin administration. Mice were injected with a single injection of vehicle (20% captisol in saline) or cisplatin (15 mg/kg IP), and kidneys and blood were harvested to detect markers of renal dysfunction. (**A**) Kidney samples from homozygous floxed stop (*Trip13*^Stop/Stop^) and *Trip13*^ΔStop^ mice were harvested from mice treated with either vehicle (-Cis; 20% captisol) or cisplatin (+Cis; 15 mg/kg IP) after 72 hours following the initial injection. Western blot analysis was performed using FLAG and GFP primary antibodies. GAPDH was used as a loading control. Arrows on the right side of the panel indicate protein standard size. (**B**) Serum creatinine and (**C**) NGAL was measured in each mouse group. (**D**) Percentage of tubular damage was determined in each group. (**E**–**H**) Representative histological images from (**E** and **G**) vehicle- or (**F** and **H**) cisplatin-treated (15 mg/kg IP) *Trip13*^Stop/Stop^ (**E** and **F**) and *Trip13*^ΔStop^ (**G** and **H**) mice. Tubular epithelial cell damage was scored as a percentage of total tubules counted. — = vehicle; + = cisplatin. *P < 0.05, ****P < 0.0001 between all other groups using 1-way ANOVA with Tukey’s post hoc analysis. *n* = 4–8 animals per group. Scale bar: 200 μm (**E**–**H**).

Cisplatin (15 mg/kg IP) was administered as a single dose into female *Trip13*^Stop/Stop^ and *Trip13*^ΔStop^ mice, and tissues were harvested after 24 or 72 hours. Protein expression for GFP and FLAG-tagged TRIP13 was clearly detectable by Western blot analysis using kidney lysates from *Trip13*^ΔStop^ mice treated with either vehicle or cisplatin (Figure 2A). No GFP or FLAG-tagged TRIP13 was detected in the *Trip13*^Stop/Stop^ mouse kidneys in either treatment group (Figure 2A).

In our initial study group, no significant difference in body weight was measured in both *Trip13*^Stop/Stop^ and *Trip13*^ΔStop^ mice at 24 (*n* = 11) and 72 hours (*n* = 6) following vehicle treatment (data not shown). At 24 hours, there was a modest 5.4% decrease in body weight after which the body weight slightly increased above the pretreatment weight by 7.4% at 72 hours. At 24 and 72 hours following cisplatin treatment, the body weight in the *Trip13*^Stop/Stop^ mice was progressively lower by 8.4% (*n* = 11) and 25.1% (*n* = 6), respectively, compared with pretreatment measurements, whereas the *Trip13*^ΔStop^ mice had a lesser decrease in body weight (13.9%) after 72 hours. The changes in body weight were associated with the extent of AKI by monitoring the blood chemistry for AKI markers, creatinine (Figure 2B) and neutrophil gelatinase-associated lipocalin (NGAL) (Figure 2C), and calculating the tubular damage (Figure 2, D–H).

Serum creatinine was significantly elevated in female *Trip13^Stop/Stop^* mice treated with cisplatin (1.95 ± 0.32 mg/dL; *n* = 6) compared with vehicle-treated littermates (0.11 ± 0.1 mg/dL; *n* = 6; Figure 2B). In the constitutive proximal tubule TRIP13-expressing (*Trip13*^ΔStop^; *n* = 8) mice following cisplatin administration, serum creatinine values were only slightly elevated (0.16 ± 0.01 mg/dL; *n* = 8) compared with the vehicle-treated mice (0.09 ± 0.01 mg/dL; *n* = 4) but significantly lower (*P* < 0.05) than those measured from the *Trip13*^Stop/Stop^ mice (*n* = 6). A similar trend was detected for serum NGAL levels, which rose to 240.0 ± 34.5 ng/L (*n* = 6) in cisplatin-treated *Trip13*^Stop/Stop^ mice but were markedly attenuated in the *Trip13*^ΔStop^ mice (Figure 2C; *n* = 8).

Histological analyses of kidney sections were scored for tubular damage in all mouse groups following treatment with a single dose of either vehicle or cisplatin (Figure 2D). Tubular damage was rare or completely nonexistent in vehicle-treated *Trip13*^Stop/Stop^ mice kidneys after 24 (*n* = 5; Figure 2D) or 72 hours (*n* = 6; (Figure 2, D and E) and *Trip13*^ΔStop^ mice kidneys after 72 hours (*n* = 4) (Figure 2, D and G). In cisplatin-treated *Trip13*^Stop/Stop^ mice, there was a significantly higher number of damaged tubules (40.1% ± 6.9%; *n* = 8; Figure 2, D and F) as determined by the presence of dilated and cast-containing tubules compared with the *Trip13*^ΔStop^ mouse kidneys, which exhibited few damaged tubules (0.4% ± 0.1%; *n* = 8; Figure 2, D and H).

### Cisplatin-dependent activation of DNA damage response pathway in mouse kidneys.

**Figure 3:**
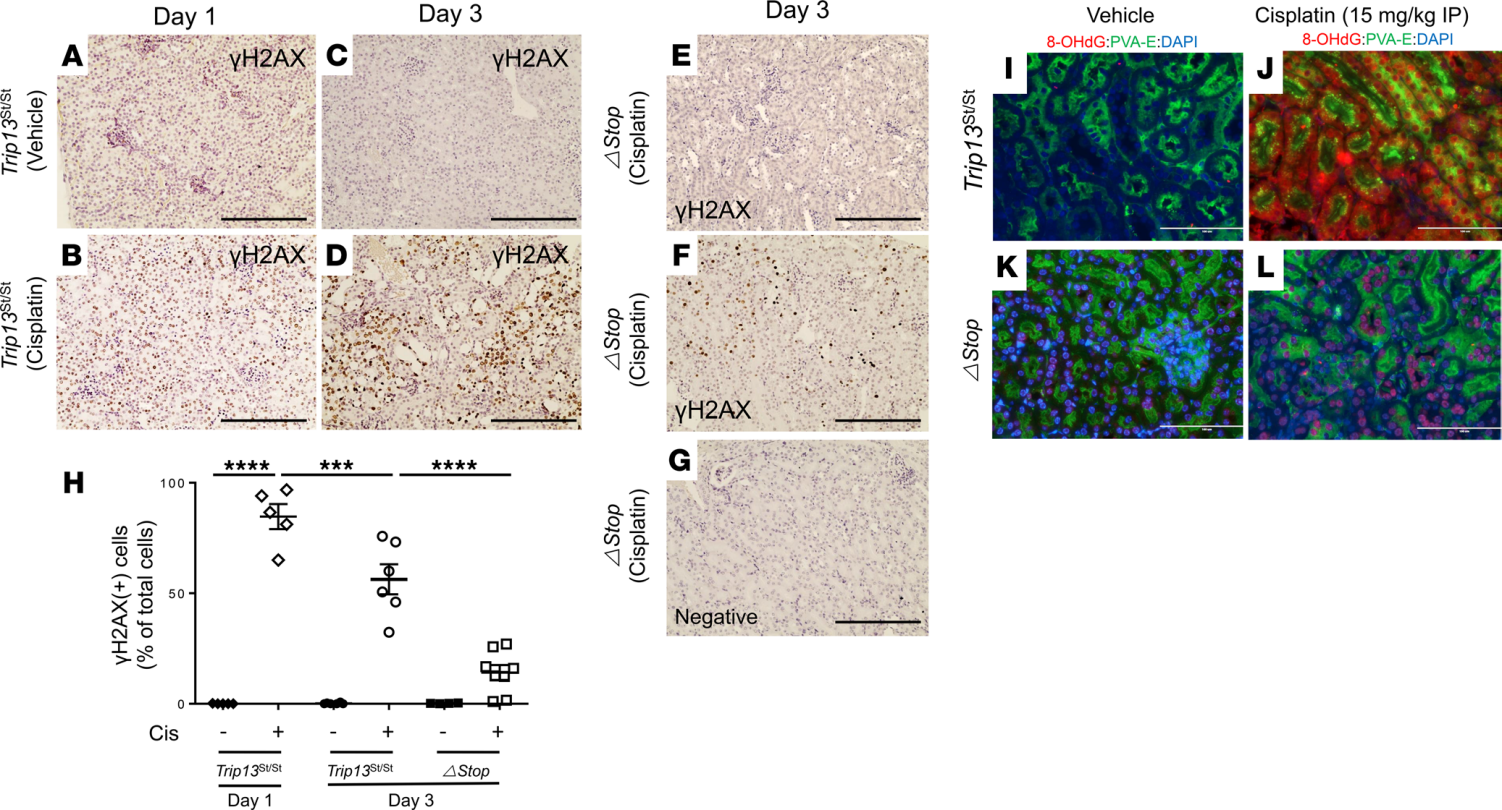
Constitutive TRIP13 overexpression in the proximal tubules reduces activation of DNA damage following cisplatin administration. Following cisplatin treatment (15 mg/kg IP), kidneys were harvested after 1 or 3 days for immunohistochemistry of (**A**–**H**) γ-H2AX (Ser139) and (**I**–**L**) 8-hydroxy-2′-deoxyguanosine (8-OHdG). After 24 (**A** and **B**) and 72 (**C**–**F**) hours following cisplatin administration, γ-H2AX (Ser139) was detected in (**A**–**D**) *Trip13*^Stop/Stop^ and (**E** and **F**) *Trip13*^ΔStop^ mice and quantified by counting positive nuclei. Sections were counterstained with hematoxylin. Negative control (no primary antibody) is shown (**G**) using sections from *Trip13*^ΔStop^ mice treated with cisplatin at day 3. (**H**) Graphical analysis of γ-H2AX (Ser139) as a percentage of total nuclei. — = vehicle; + = cisplatin. ***P < 0.001, ****P < 0.0001 between the indicated groups using 1-way ANOVA with Tukey’s post hoc analysis. (**I**–**L**) Immunofluorescence of 8-OHdG in kidney sections from vehicle- and cisplatin-treated *Trip13*^Stop/Stop^ and *Trip13*^ΔStop^ mice after day 3. Alexa Fluor 555 (red) fluorescence was used to detect 8-OHdG, and Alexa Fluor 488 (green) fluorescence was used to detect proximal tubule lectin (PVA-E). DAPI was used to detect nuclei (blue). Scale bar: 200 μm (**A**–**G**); 100 μm (**I**–**L**). *n* = 4–8 animals per group.

Phosphorylated H2AX (γ-H2AX; Ser139) was detected at 24 and 72 hours after cisplatin treatment, indicating DNA damage response activation (Figure 3). Renal tubular epithelial cells positive for γ-H2AX were rarely detected in vehicle-treated *Trip13*^Stop/Stop^ mouse kidneys at both 24 (Figure 3, A and H) and 72 hours (Figure 3, E and H; 0.1%–0.2%; *n* = 5–6/time point). At 24 hours, cisplatin-treated *Trip13*^Stop/Stop^ mice had a high number (84.7% ± 5.6%; *n* = 5) of the tubular epithelial cells positive for γ-H2AX (Figure 3, B and H). At 72 hours, the number of γ-H2AX–positive tubular epithelial cells decreased from day 1 but remained elevated (56.3% ± 7.5%; *n* = 6) in the cisplatin-treated *Trip13*^Stop/Stop^ mice (Figure 3, D and H). In the *Trip13*^ΔStop^ mice, there was a significantly lower percentage of γ-H2AX–positive tubular epithelial cells (14.0% ± 4.0%; *P* < 0.05; *n* = 8) following treatment with cisplatin after 72 hours (Figure 3, F and H). Rare activation of γ-H2AX was detected (0.2% ± 0.1%; *n* = 4) in vehicle-treated *Trip13*^ΔStop^ mouse kidneys (Figure 3, E and H). No γ-H2AX–positive tubular epithelial cells were detected in the negative control section without the antibody (Figure 3G).

To determine whether other nucleotide modifications were observed following cisplatin administration, we performed immunofluorescence on the kidney sections for 8-hydroxy-2′-deoxyguanosine (8-OHdG), which is a common variant observed during oxidative stress–related damage. At 72 hours, 8-OHdG was readily detected in the cisplatin-treated *Trip13*^Stop/Stop^ mouse kidneys (Figure 3J), whereas no nuclei were positive for 8-OHdG in the vehicle-treated mouse littermates (Figure 3I). In the *Trip13*^ΔStop^ mice, there was no detection of 8-OHdG in the vehicle-treated kidneys (Figure 3K) and considerably less intense fluorescence for 8-OHdG–positive nuclei following cisplatin treatment (Figure 3L).

**Figure 4:**
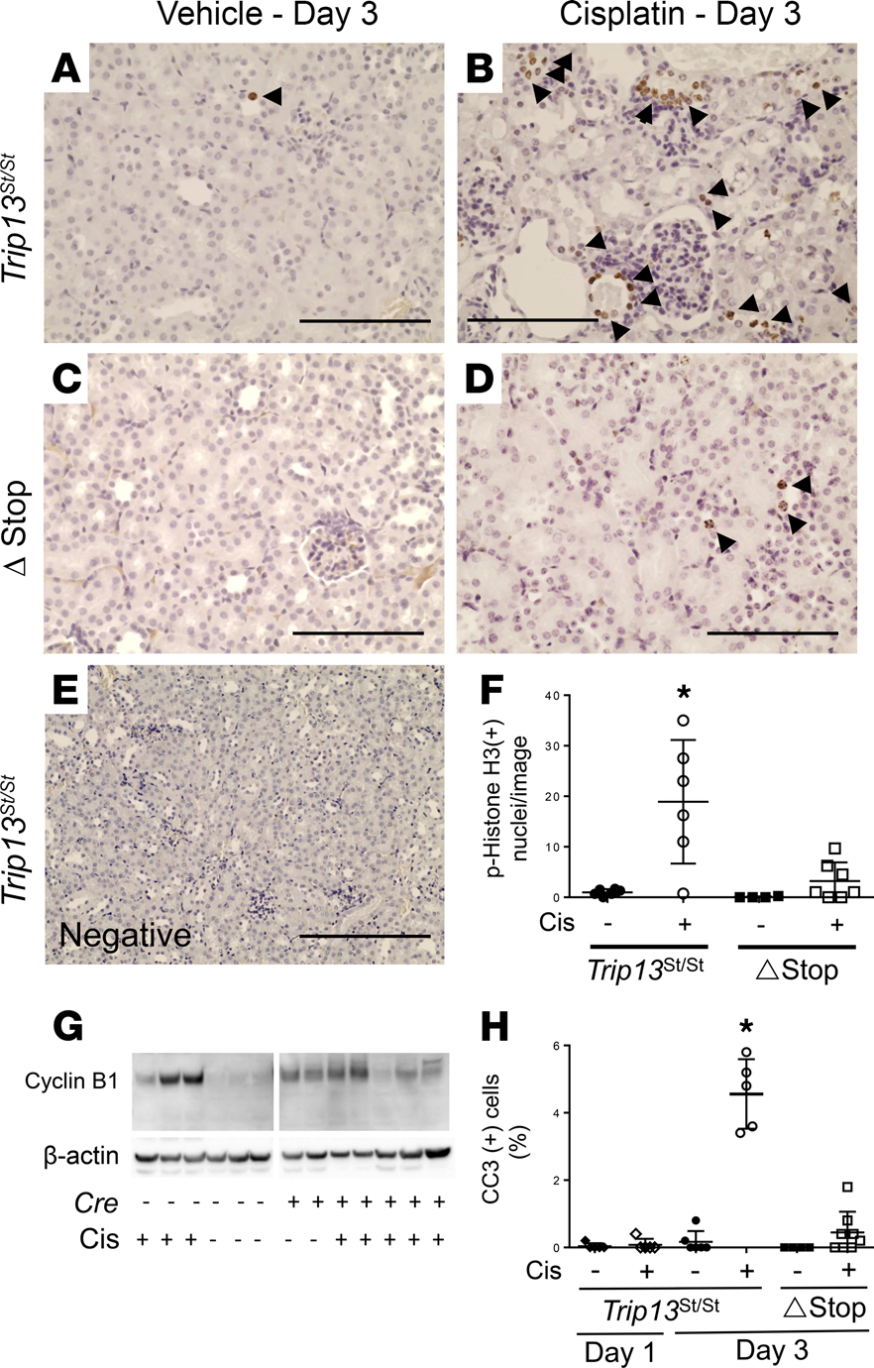
Constitutive TRIP13 overexpression reduces apoptotic signaling and regulates intracellular signaling pathways. (**A**–**E**) Representative images are shown for p–histone H3 (Ser10) in kidney sections obtained from vehicle- and cisplatin-treated *Trip13*^Stop/Stop^ (**A** and **B**) and *Trip13Δ*^Stop^ (**C** and **D**) mice after day 3. Arrowheads are pointing at individual or clusters of stained nuclei. (**E**) A negative control (no primary antibody) section was used from a cisplatin-treated *Trip13*^Stop/Stop^ mouse. (**F**) Graphical analysis showing quantitative numbers of nuclei positive for p–histone H3 (Ser10) in each image (3–4 images were counted per kidney section in each mouse). (**G**) Cyclin B1 bands were shown following Western blot analysis using kidney lysates isolated from *Trip13*^Stop/Stop^ and *Trip13Δ*^Stop^ mice treated with either vehicle or cisplatin (15 mg/kg IP) after 72 hours. (**H**) Cleaved caspase-3–positive cells were counted relative to the total number of nuclei. — = vehicle; + = cisplatin (15 mg/kg IP). *P < 0.01 between all groups using 1-way ANOVA with a Tukey’s post hoc analysis. Scale bar: 100 μm (**A**–**D**), 200 μm (**E**). *n* = 4–8 animals/group.

The temporal activation of γ-H2AX following cisplatin treatment was associated with increased G2/M blockade using phosphorylated (p-) histone H3 (Ser10; Figure 4, A–F), increased accumulation of cyclin B1, and proapoptotic signaling by detecting cleaved caspase-3–positive tubular epithelial cells (Figure 4H). More specifically, the number of p–histone H3–positive (Ser10-positive) nuclei in tubular epithelia was significantly higher (*P* < 0.01) in cisplatin-treated *Trip13*^Stop/Stop^ mice (18.9 ± 5.0 nuclei/image; *n* = 6; Figure 4, B and F) compared with both the vehicle-treated *Trip13*^Stop/Stop^ (1.0 ± 0.3 nuclei/image; *n* = 6; Figure 4, A and F) and *Trip13*^ΔStop^ (1.1 ± 0.6 nuclei/image; *n* = 4; Figure 4, C and F) mouse groups. In the cisplatin-treated *Trip13*^ΔStop^ mice, there was a significantly (*P* < 0.01) lower number of p–histone H3–positive (Ser10-positive) cells (2.8 ± 1.5/image; *n* = 8; Figure 4, D and F) compared with vehicle-treated littermates. No positive cells were detected in the negative control (no antibody) sections (Figure 4E).

The increase in G2/M-positive tubular epithelial cells at 72 hours after cisplatin treatment was associated with a considerable increase in band intensity of cyclin B1 (Figure 4G) compared with vehicle treatment in the *Trip13*^Stop/Stop^ kidneys. In the *Trip13*^ΔStop^ kidney lysates, endogenous levels of cyclin B1 were elevated under normal conditions with no significant difference in the cyclin B1 accumulation in the cisplatin-treated *Trip13*^ΔStop^ kidney lysates.

We found asignificantly higher percentage (4.5% ± 0.9%; *n* = 5; *P* < 0.0001) of cleaved caspase-3–positive nuclei in cisplatin-treated *Trip13*^Stop/Stop^ mice compared with the proximal tubular TRIP13-overexpressing *Trip13*^ΔStop^ mouse kidneys (0.5% ± 0.2%; *n* = 7) (Figure 4H). Rare positive cells for cleaved caspase-3 were evident in vehicle-treated *Trip13*^Stop/Stop^ kidneys at both 24 (0.04% ± 0.02%; *n* = 5) and 72 hours (0.2% ± 0.1%; *n* = 6), or 24 hours following cisplatin treatment (0.08% ± 0.1%; *n* = 5; Figure 4H). Tubular epithelial cell proliferation was determined by staining with Ki67 and/or proliferating cell nuclear antigen (PCNA) staining (Supplemental Figure 1; supplemental material available online with this article; https://doi.org/10.1172/jci.insight.139092DS1). There was a significantly higher percentage of Ki67-positive nuclei by ~3.2-fold (*P* < 0.001) in the cisplatin-treated *Trip13*^Stop/Stop^ mouse kidneys (3.7% ± 0.5%; *n* = 4) compared with the vehicle-treated *Trip13*^Stop/Stop^ group (1.2% ± 0.1%; *n* = 6). In the vehicle-treated *Trip13*^ΔStop^ mouse kidneys where Cre activation induced selective proximal tubular *Trip13* expression, the percentage of Ki67-positive cells (1.1% ± 0.2%; *n* = 4) was not significantly different from the control Cre-minus *Trip13*^Stop/Stop^ mouse kidneys. Cisplatin treatment produced a small, but insignificant, increase in the Ki67-positive nuclei from the *Trip13*^ΔStop^ mouse kidneys (1.6% ± 0.2%; *n* = 8). A similar profile in PCNA-positive nuclei was observed in the same kidneys (Supplemental Figure 1).

In male mice, cisplatin at the same dose administered to females tended to have a slower and less potent effect on body weight and kidney damage (Supplemental Figure 2). Serum creatinine was significantly lower (*P* < 0.001) at 0.8 ± 0.2 mg/dL (*n* = 8) in males compared with 1.95 mg/dL (*n* = 6) in females (Figure 2B) after 72 hours. Similarly, γ-H2AX (24.9% ± 1.9%; *n* = 8) was significantly lower (*P* < 0.0001) by more than 50% in males compared with the females (56.3% ± 7.5%; Figure 3H), and this was associated with less tubular damage (29.4% ± 5.0%; *n* = 8) by approximately 25% than females (Figure 2D).

### Blockade of double-stranded DNA repair exacerbates cisplatin-dependent tubular damage.

To evaluate whether the recovery of DNA damage associated with cisplatin nephrotoxicity required double-stranded DNA break repair pathway activation, selective inhibitors to homologous recombination (mirin) or NHEJ (NU7441) were administered to determine the effects on tubular damage and renal function.

**Figure 5:**
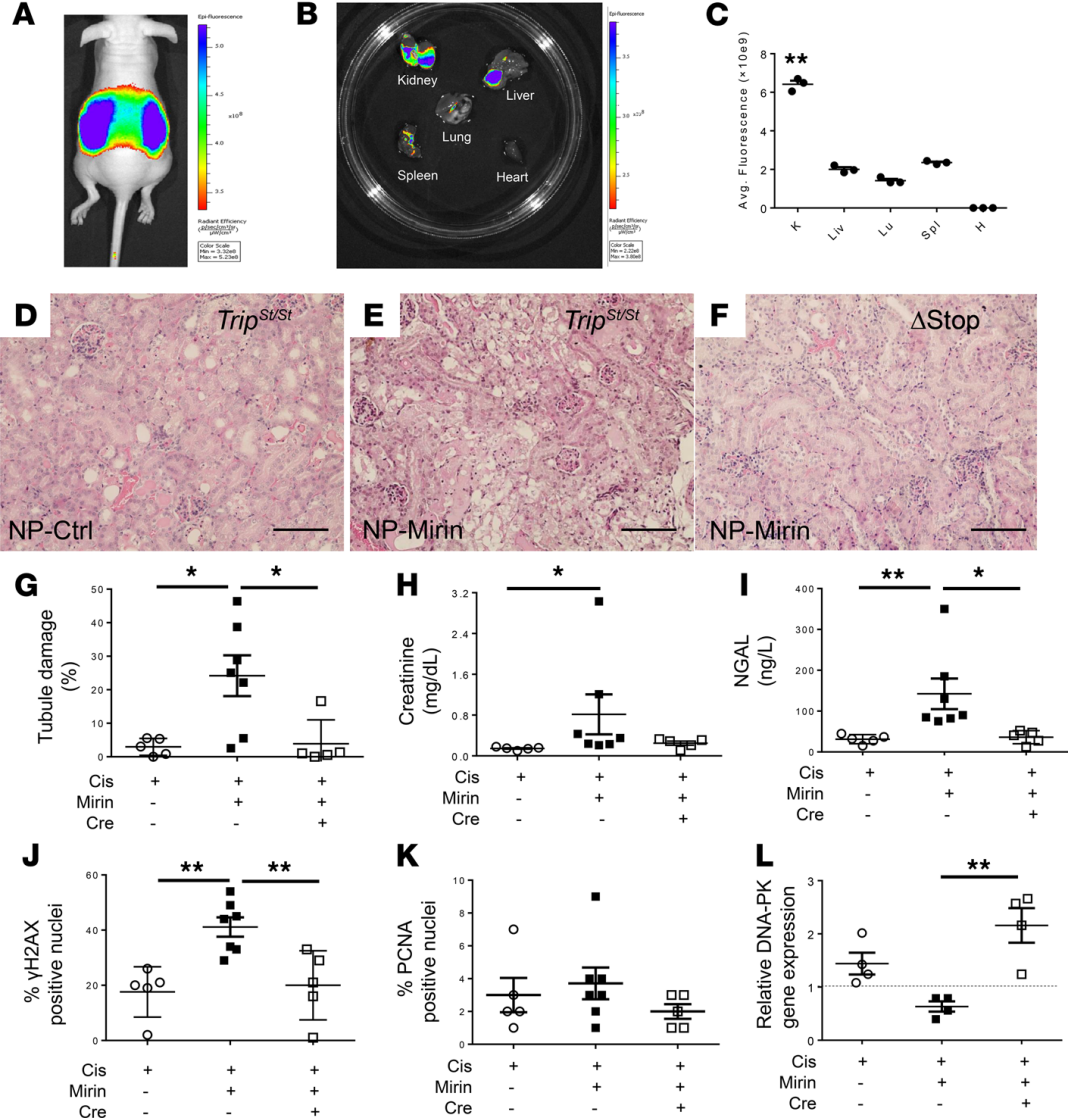
Inhibition of double-strand break repair promotes tubular epithelial cell damage. (**A** and **B**) Nanoparticles (50 μg) containing fluorescent ICG dye were injected into mice and monitored 24 hours later using IVIS system in (**A**) living mice and (**B**) ex vivo excised organs. (**C**) Ex vivo organ fluorescence accumulation (*n* = 3 animals). K, kidney; Lu, lung; Liv, liver; H, heart; Spl, spleen. Data represent mean ± SD. (**D**–**G**) Representative histological images from (**D**) vehicle- or (**E** and **F**) cisplatin-treated (15 mg/kg IP) *Trip13*^Stop/Stop^ (**D** and **E**) and *Trip13Δ*^Stop^ (**F**) mice. (**G**) Tubular epithelial cell damage was scored as a percentage of total tubules counted. Serum markers of AKI were monitored for (**H**) creatinine and (**I**) NGAL after 72 hours following treatment with cisplatin (15 mg/kg IP) and NP-mirin or NP-Ctrl (50 mg/kg IP). (**J**) γ-H2AX– (Ser139) and (**K**) PCNA-positive nuclei in kidney sections at 72 hours following cisplatin treatment with and without mirin. NP without mirin (50 mg/kg IP) was used as the control solution. (**L**) Relative gene expression change following treatment with cisplatin and/or mirin. Each of the respective groups was compared with mouse kidney values obtained from control vehicle-treated *Trip13*^Stop/Stop^ mice. (**G**–**K**) *n* = 5–7 mice/group; (**L**) *n*=4 mice/group. *P < 0.05, **P < 0.01 between all organs (**C**) or indicated groups (**G**, **I**, **J**, and **L**) or all groups (**C**) using 1-way ANOVA with Tukey’s post hoc analysis. Significance for creatinine (**H**) was determined using Kruskal-Wallis nonparametric test with Dunn’s post hoc analysis. Scale bar: 100 μm. + = administration of cisplatin or mirin; Cre - = *Trip13*^Stop/Stop^; Cre + = *Δ*Stop.

Mirin is a selective inhibitor of Mre11-mediated homologous recombination (27), with relatively poor solubility for in vivo studies. To improve the bioavailability of mirin, a nanoparticle formulation was prepared similarly to a prior study (28) and injected into the mice (50 mg/kg IP) immediately following the injection of cisplatin (15 mg/kg IP). As a control, nanoparticles devoid of mirin were injected in a separate group of mice. The chemical properties of the nanoparticles are shown in Supplemental Figure 3. As a proof of concept, the nanoparticle formulation (50 μg) with indocyanine green (ICG) dye demonstrated predominantly strong signal in intact mouse kidneys (Figure 5A) and following organ harvesting (Figure 5B) after 24 hours following IP administration. Figure 5C provides quantitative fluorescence in the isolated organs of the injected mice after 24 hours, which showed that the kidney was about 4 times higher in fluorescence compared with the other organs, including the liver, lung, spleen, and heart.

Using the nanoparticle formulation in cisplatin-treated male *Trip13*^Stop/Stop^ mice, the nanoparticle formulation containing mirin (NP-Mirin) exerted significantly increased (*P* < 0.05) tubular damage (Figure 5, D–G) and serum markers of AKI (creatinine and NGAL) (Figure 5, H and I) after 72 hours compared with their control littermates treated with nanoparticles (NP-Ctrl). In this group, the lower creatinine levels following cisplatin treatment on day 3 are probably attributed to a combination of the use of males rather than females as well as the relatively high volume of NP solution (~0.7–1 mL) needed for injection into the mice. Consistent with the increased physical damage and serum markers of AKI, γ-H2AX–positive (Ser139-positive) cells were significantly higher (*P* < 0.01) in the NP-Mirin (40.9% ± 3.5%; *n* = 7) versus NP-Ctrl group (17.5% ± 4.0% cells; *n* = 5) (Figure 5J). Overexpression of proximal tubular TRIP13 prevented the tubular damage even in the presence of mirin, and this was associated with lower serum creatinine (0.23 ± 0.04 mg/dL; *P* < 0.05), lower NGAL measurement (38.1 ± 8.5 ng/mL; *n* = 5; *P* < 0.05), and fewer γ-H2AX–positive cells (20.0% ± 5.8%; *n* = 5; *P* < 0.05). No statistical difference was calculated between the different groups for PCNA-positive nuclei regardless of the TRIP13 status (Figure 5K).

In addition, in the mice treated with both mirin and cisplatin, there was a significant ~2-fold increase (*P* < 0.01; *n* = 4 mice/group) in DNA-PKcs mRNA levels compared with vehicle-treated mice (Figure 5L; *n* = 4/group), which suggests a potential effect by the NHEJ pathway to compensate for DNA repair when the homology directed repair pathway is partially to completely blocked.

To evaluate the role of the NHEJ pathway during cisplatin injury, a selective inhibitor to the DNA-PKcs, NU7441 (20 mg/kg IP), was tested in a separate group of male *Trip13*^Stop/Stop^ mice. The experimental time was shortened compared with the other groups because of the 30% mortality rate that was occurring within 48 hours after treatment with both cisplatin and NU7441 in the *Trip13*^Stop/Stop^ mice. All other non-NU7441-treated groups only exhibited observations of morbidity, but not mortality, prior to day 3 following cisplatin treatment.

**Figure 6:**
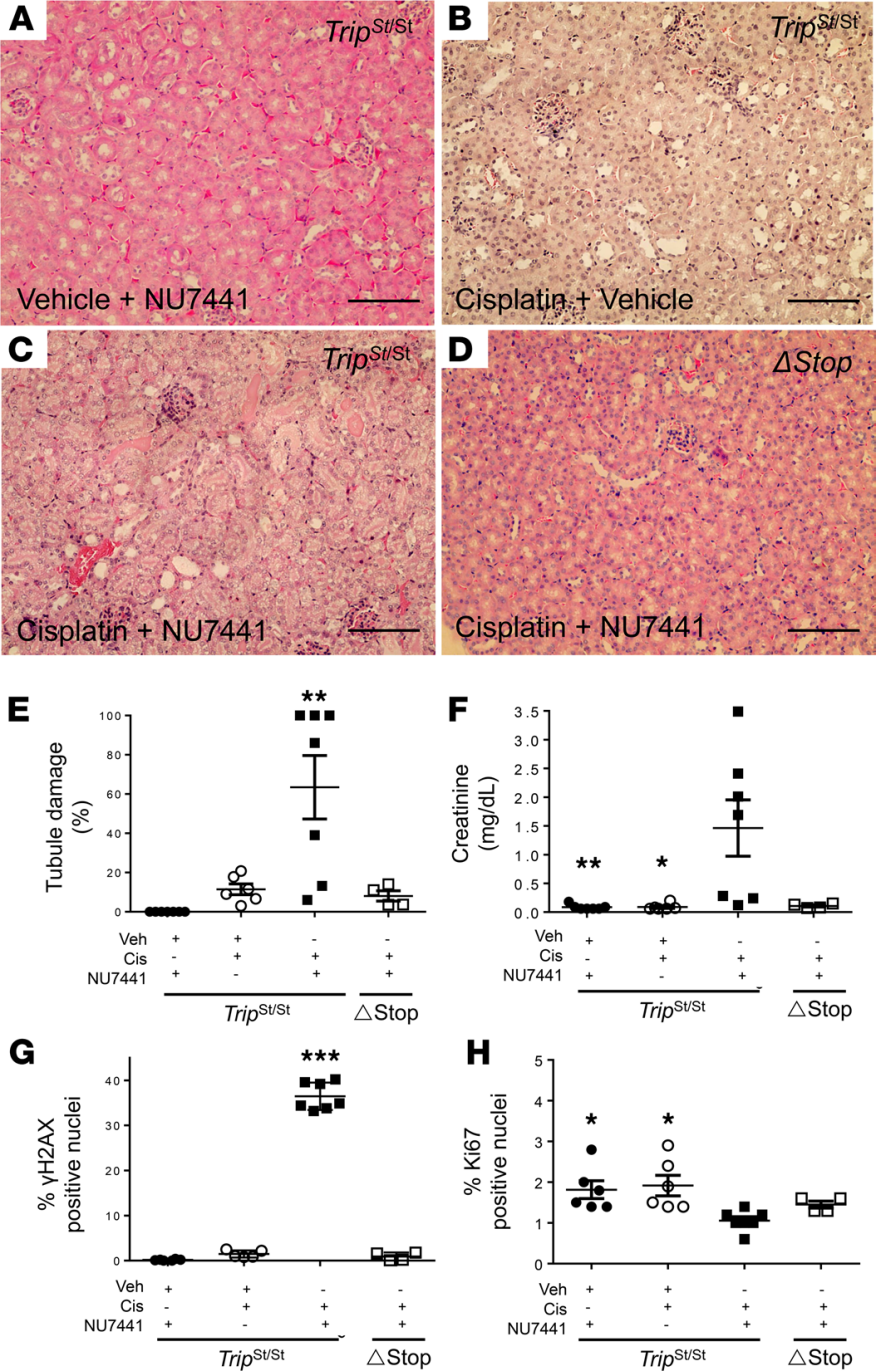
Inhibition of DNA-PKcs accelerates AKI. NU7441 (20 mg/kg) was administered IP to *Trip13*^Stop/Stop^ and *Trip13Δ*^Stop^ mice that were euthanized prior to or at 48 hours to collect blood and kidneys. (**A**–**D**) Representative histological images from (**A**) vehicle- or (**B**–**D**) cisplatin-treated (15 mg/kg IP) *Trip13*^Stop/Stop^ (**A**–**C**) and *Trip13Δ*^Stop^ (**D**) mice coadministered with either vehicle (**B**) or NU7441 (20 mg/kg IP; **A**, **C**, and **D**). (**E**) Tubular epithelial cell damage was scored as a percentage of total tubules. **P < 0.01 between all groups. (**F**) Serum markers of AKI were measured for creatinine after 48 hours following treatment with cisplatin (15 mg/kg IP). *P < 0.05; **P < 0.01 between cisplatin- and NU7441-treated mice. Positive nuclei for (**G**) γ-H2AX (Ser139) and (**H**) Ki-67 in kidney sections at 48 hours following cisplatin treatment with and without NU7441. *P < 0.05 between both genetic strains treated with cisplatin- and NU7441; ***P < 0.001 between all groups. One-way ANOVA with Tukey’s post hoc analysis was performed for each set of data (**E**–**H**). *n* = 4–7 mice/group. Scale bar: 100 μm. *Δ*Stop, *Trip13Δ*^Stop^ mice; *Trip13*^St/St^, *Trip13*^Stop/Stop^ mice.

Administration of NU7441 with vehicle solution for cisplatin (~0.1 mL) was injected at time 0 and at 24 hours, and the mouse kidneys were harvested after 48 hours. No adverse change in tubular architecture (Figure 6, A–E) or renal function (0.09 ± 0.02 mg/dL; *n* = 7; Figure 6F) was observed. Following treatment with cisplatin (15 mg/kg IP), the NU7441-treated *Trip13*^Stop/Stop^ mice had significantly higher (*P* < 0.001) serum creatinine (1.47 ± 0.49 mg/dL; *n* = 7) after 48 hours compared with cisplatin-treated littermates without NU7441 (0.07 ± 0.01 mg/dL; *n* = 6). Tubular damage was consistent with the serum creatinine values where the NU7441-treated mice exhibited significantly higher tubular damage (63.5% ± 16.1%; *P* < 0.01; *n* = 7) compared with mice only treated with cisplatin and vehicle (9.8% ± 2.8%; *n* = 7) or vehicle alone (*n* = 7) (Figure 6, A–E). Similarly, there was a tremendously higher number of γ-H2AX–positive nuclei (Figure 6G) in the cisplatin-treated *Trip13*^Stop/Stop^ with (36.5% ± 1.2%; *n* = 7) or without NU7441 (1.5% ± 0.4%; *n* = 7). Minimal activation of γ-H2AX was detected in control *Trip13*^Stop/Stop^ mice treated only with NU7441 (0.2 ± 0.1; *n* = 7). In the *Trip13*^ΔStop^ mice (*n* = 4), cisplatin with NU7441 treatment did not lead to any measurable change in serum creatinine (0.11 ± 0.02 mg/dL) (Figure 6F) or γ-H2AX–positive nuclei (1.8% ± 0.8%; *n* = 4) (Figure 6G) and only 9.5% ± 2.0% (*n* = 4) of tubular damage after 48 hours (Figure 6, D and E). In cisplatin-treated *Trip13*^Stop/Stop^ mice, treatment with NU7441 led to a small, but significant (*P* < 0.05), reduction in Ki67-positive nuclei (1.0% ± 0.1%; *n* = 6) compared with vehicle-treated mice (1.9% ± 0.3%; *n* = 6) (Figure 6H). No significant difference in Ki67-positive nuclei was calculated between cisplatin-treated *Trip13*^Stop/Stop^ (1.0% ± 0.1%; *n* = 7) and *Trip13*^ΔStop^ (1.5% ± 0.1%; *n* = 4) mice treated with NU7441. In the absence of cisplatin, *Trip13*^Stop/Stop^ mice treated only with NU7441 had a modest, but significantly higher, number of Ki67-positive nuclei (*P* < 0.05; 1.8% ± 0.2%; *n* = 6).

## Discussion

In the present study, constitutive de novo overexpression of TRIP13 in proximal tubules provided considerable protection to the kidney from acute nephrotoxic effects of cisplatin. Even though cisplatin or other platinum-based compounds remain commonly prescribed to combat various types of cancer because of their continued effectiveness, with constant daily use, these drugs can accumulate in the proximal tubules and often produce deleterious acute effects in the kidney.

One of the major consequences of cisplatin accumulation is the formation of reactive oxygen species leading to genomic DNA modifications at the nucleotide level that can even produce lethal physical breaks in the DNA. Consistent with this finding, we observed increased fluorescent nuclei for 8-OHdG, which is a biomarker for oxidative damage (29) and can be an indicator for clustered DNA lesions that could indicate double-stranded DNA breaks (DSBs) (30). Another early response to the DNA damage, including DSBs, is the phosphorylation of a histone variant H2A (γ-H2AX) by distinct sensors (31), such as ataxia telangiectasia mutated (ATM) (32, 33), which is a primary mobilizer of the cellular response to DSBs. Due to the pleiotropic effects induced by cisplatin on DNA, other sensors may be activated by crosstalk mechanisms with ATM, including ataxia telangiectasia and Rad3 related (ATR) and DNA-PK, to control not only the phosphorylation of H2AX (34), but also other downstream transducers (35). In general, a majority (about two-thirds) of the cells that are destined to survive progress through the S phase of the cell cycle and exit mitosis. This would lead to the formation of new and properly oriented epithelial cells (36, 37). To achieve this level of recovery, the fate of the surviving tubular epithelial cells is determined by arresting the cell cycle at distinct points, such as the intersection of G1/S and G2/M stage. This enables cells to make a decision on whether to activate pathways to restore normal cellular function and become mitotically active or undergo apoptotic cell death (38, 39). However, prolongation of G2/M arrest, which occurs in various forms of AKI, can tilt the balance of the cellular fate toward cell death (40). This would inevitably slow or produce incomplete recovery and lead to further deterioration in renal function (38, 39).

At present, the upstream signaling pathways involved with TRIP13 during DNA damage repair in the mammalian system have yet to be fully described. In cultured tubular epithelia, cisplatin preferentially activates the ATR/Chk2 signaling axis to promote renal cell apoptosis through TP53 activation (41). More recently, proximal tubule knockout of ATR resulted in increased tubular damage, DNA damage, fibrosis, and a loss of kidney function following kidney injury, which was associated with increased production of p53 and p21 (42). In oocytes with reduced endogenous production of TRIP13, partial activity of TP53 and its paralog, TAp63, was detected, leading to increased apoptotic cell death due to incomplete double-stranded DNA repair (25). A previous study from our lab also showed that injured tubular epithelial cells could activate phosphorylated forms of TP53 (26), and this mechanism was linked to a direct interaction between TRIP13 and tetratricopeptide 5, which is a coactivator of p53 (26). Depending upon the type and extent of DNA damage, TRIP13 may be a key regulator in controlling the pathways involved in DNA repair and cell cycle progression, especially since hypomorph TRIP13 mice were unable to completely repair their germline DNA, resulting in their fate being directed toward apoptosis (25). Further studies are needed to explore how sensors and effectors normally associated with dSBs, namely ATM and DNA-PKcs, are involved with TRIP13.

With that said, TRIP13 overexpression has been shown to promote a more aggressive state of growth in somatic head and neck cancer cells by activating double-stranded DNA repair through the NHEJ pathway (23). In that study, TRIP13 promoted DSB repair by directly binding and activating DNA-PKcs (23). In general, NHEJ is considered the predominant pathway by which DSBs are repaired, particularly in lowly mitotic cells (43). However, other pathways exist, such as homologous recombination, to ensure high-fidelity repair following DNA damage. Emerging data that suggest TRIP13 acting as an AAA^+^ ATPase can remodel protein conformations to actively switch the specific pathway involved in double-stranded DNA repair. For example, Rev7-Shieldin complexes, which normally activate NHEJ pathways, can be dissociated by TRIP13 through a conformational change in Rev7 (44–46). This leads to a transition where the double-stranded DNA repair is performed by homology-dependent recombination instead of NHEJ (44–46). A similar switch to homologous recombination occurs when TRIP13 dissociates Rev3-Rev7 complexes, which normally activate the translesion synthesis (TLS) pathway (44, 47). In our study, TRIP13 overexpression in the proximal tubules may have been able to interchangeably swap DNA repair pathways following cisplatin injury in the presence of selective pathway inhibitors, which led to the limited tubular damage and preserved kidney function.

In addition to DNA repair, TRIP13 is involved in the regulation of the mitotic checkpoint complex (MCC) (17–21, 48–51), which comprises a heterotetrameric protein complex that functions as the primary inhibitor of the spindle assembly checkpoint (SAC). The SAC process maintains genome stability by delaying anaphase until metaphase chromosomes are properly aligned with spindle attachment (52). In the case of cisplatin treatment, damaged kidney cells may be arrested at different phases of the cell cycle, including G2/M, where the MCC would form and bind to the anaphase-promoting complex or cyclosome (APC/C) to inhibit the SAC. To disassemble the MCC, TRIP13 performs a series of mechanistic steps, which includes binding to p31^comet^ and hydrolyzing ATP through its ATPase activity, to facilitate a conformational change in a key MCC protein, mitotic arrest defective protein 2 (Mad2), from an active closed (C-Mad2) to an inactive open (O-Mad2) state (17–21, 48–51). This is an important regulatory step as the formation of an active MCC is dependent upon the interaction of C-Mad2 with CDC20, which is part of the MCC that binds to APC/C and prevents SAC activation. In vitro experiments have shown that the TRIP13 is only capable of disassembling free MCC that is not bound to the APC/C (51). This context-dependent effect by TRIP13 may be important during conditions of mitotic arrest, where TRIP13 levels were essential in determining the length of mitosis; i.e., in the absence of TRIP13, mitotic length was considerably reduced whereas overexpression of TRIP13 rescued the mitotic length of the cell (18). Unperturbed mitosis is not dependent on the regulatory actions of TRIP13 (18). Another factor that could affect both DNA repair and mitotic progression is the potential competition in binding p31^comet^ by either homodimer Rev7 or Mad2. The interaction by p31^comet^ with Rev7 instead of Mad2 could affect mitotic exit by dysregulating the control of the SAC by MCC. Alternatively, p31^comet^ sequestration of Rev7 from its normal binding partner, Rev3, could limit the ability of the damaged DNA to recover using the TLS pathway following cisplatin exposure (53). This may partially explain some of our observations where TRIP13 overexpression led to an increased accumulation of cyclin B1 in normal kidneys. A constant pool of inactive O-Mad2 may have been produced from C-Mad2, which would have led to the dissociation of the non-APC/C-bound MCC into its individual constituents. Upon exposure to an injurious stimuli, like cisplatin, the mitotic length may not have been negatively affected or even blocked due to the absence of free MCC. This may have enabled a higher number of injured cells to continue with their DNA repair, especially being potentiated by TRIP13 overexpression. In the end, this may have directed the fate of the initially damaged cell toward survival rather than death due to the more efficient repair of the damaged DNA.

In conclusion, our study demonstrated the production and testing of a floxed stop (LSL) mouse model that can selectively induce *Trip13* expression for in vivo investigation in the kidney, and potentially for other organs in normal and injury states. Here, we present data that constitutive overexpression of TRIP13 in the proximal tubules following cisplatin administration prevented the increased phosphorylation of H2AX in conjunction with reduced blockade at the G2/M phase of the cell cycle. It would appear that normal tubular epithelia are in a higher state of mitotic activity presumably due to TRIP13 overexpression in vivo. This may have promoted cell survival and limited apoptotic cell death from the nephrotoxic effects of cisplatin. Since TRIP13 was detected in normal and AKI human kidney sections (Supplemental Figure 4) similar to our findings in mice (26), TRIP13 or its associated binding proteins may become potential therapeutic targets to protect the kidney from not only cisplatin nephrotoxicity but also possibly other forms of acute and chronic kidney disease.

## Methods

### Generation of conditional transgenic mice to express Trip13.

Conditional *Trip13*-transgenic mice were generated using a floxed stop strategy (Cyagen). The targeting construct was designed (from 5′ to 3′) as follows: a 5′ homology arm, a ubiquitously expressed CAG hybrid promoter, a *loxP*-flanked 3xSV40 poly(A) stop cassette, a 3X-FLAG epitope tag fused to Trip13, a T2A ribosomal skip cleavage peptide sequence, an EGFP gene, a bovine growth hormone poly(A), and a 3′ homology arm. The mouse expressing γ-glutamyl transpeptidase–Cre recombinase mouse was bred into the homozygous lox-Stop-lox-*Trip13-GFP* (*Trip13*^Stop/Stop^) mouse to produce the conditional GGT1-Cre^+/–^ *Trip13*-GFP (*Trip13*^*Δ*Stop^) mice. Mice were housed, bred, and maintained in a specific pathogen–free animal facility at the University of Tennessee Health Science Center. All experiments were approved by the IACUC (see *Study approval*). Mice were allowed ad libitum access to food and water and maintained in a 12-hour light/12-hour dark cycle. Genotypes were confirmed by PCR amplification of genomic DNA isolated from toe or tail snip. The conditions for the floxed primers (F1/R1 and F2/R2) were as follows: 94°C for 3 minutes; then 32 cycles of 94°C for 30 seconds, 66°C for 30 seconds, and 72°C for 12 seconds; and then a final extension of 72°C for 7 minutes. To detect Cre recombinase, the PCR conditions for Cre for/Cre rev were as follows: 94°C for 3 minutes; then 32 cycles of 94°C for 30 seconds, 60°C for 30 seconds, and 72°C for 5 seconds; and then a final extension of 72°C for 7 minutes. The PCR product sizes were as follows: F1/R2 = 616 bp; F2/R2 = 453 bp; Cre for/Cre rev = 204 bp. We used an initial pair of GGT1-Cre primers shown in Figure 1B, which were 400 bp in size. PCR primer sequences are listed in Table 1.

### Murine model of cisplatin-induced nephrotoxic AKI.

Cisplatin nephrotoxic AKI was induced in randomly distributed male and female *Trip13*^Stop/Stop^ and GGT1-Cre^+/–^ *Trip13^Stop/Stop^* mice (aged 8–12 weeks). Water was removed from the mice for at least 12 hours prior to injecting the mice with a single IP dose of cisplatin (15 mg/kg) (Advanced ChemBlocks, catalog K12017) or vehicle (20% captisol dissolved in sterile saline). In some mice, mirin (50 mg/kg) or NU7441 (20 mg/kg) was injected into the IP space either immediately or 6 hours after the administration of cisplatin, respectively. Body weights were measured prior to the injection and every 24 hours for the duration of the experimental period. Blood was collected by submandibular bleeding after 24 and 72 hours following the initial injection to isolate plasma for measurement of creatinine by liquid chromatography–tandem mass spectrometry (Department of Biochemistry, University of Alabama at Birmingham) and lipocalin-2 by ELISA kit (NGAL; Abcam ab119601). Prior to euthanasia, kidneys were removed and placed either in neutral buffered formalin for paraffin embedding or on dry ice for protein isolation.

### Chemicals and reagents for NP formulation.

Poly(D, l-lactide-*co*-glycolide) (PLGA) (50:50 lactide-glycolide ratio, MW: 31,000–50,000, ester terminated) was purchased from Birmingham Polymers. Poly(vinyl alcohol) (PVA) (363138, MW: 30,000–70,000), poly(l-lysine) (PLL) (MW: 30,000–70,000), Pluronic F-68 (P1300, MW: 8350), and coumarin 6 (442631, 98%) were purchased from MilliporeSigma and used without further purification.

### Preparation and characterization of PLGA-mirin NPs.

PLGA NP formulation with mirin and control PLGA NPs were prepared by nanoprecipitation technique (54). Briefly, PLGA (90 mg) with or without mirin (50 mg) was dissolved in 10 mL of acetone to achieve a uniform solution; 2% PVA-aqueous solution was prepared in cold water, kept under stirring until it dissolved. To this freshly prepared PVA solution, PLGA-mirin or PLGA-alone hydrophobic solution was added dropwise and stirred on a magnetic stir plate at 1000 rpm. The next day, after the acetone evaporated, 10 mg of PLL and 10 mg of Pluronic F-68 were dissolved in water, added to the NP suspension, and stirred for at least 6–8 hours. The formulation was stored at 4°C until further use. Similarly, ICG-PLGA NPs were prepared by replacing mirin for mouse imaging studies (55, 56).

The particle size (nm) and zeta potential (mV) of prepared PLGA NPs and PLGA-mirin NPs were acquired by dynamic light scattering principle using Zetasizer (Nano ZS, Malvern Instruments) (57). The particle size measurements were performed at 25°C using 50 μL of freshly prepared PLGA NPs diluted in 1 mL of ultrapure water and probe sonicated using VirSonic Ultrasonic Cell Disrupter 100 (The VirTis Company) for 30 seconds. Particle size of formulations was measured in filtered water/PBS/DMEM to confirm stability of the particle in a physiologically relevant environment. The measurement was reported from 3 runs (~2 minutes each run). The zeta potential of NPs (50 μL of nanoformulation in 1 mL of 1× PBS) was measured in triplicate (each reading = 30 runs) by laser Doppler microelectrophoresis technique. Fourier transform infrared (FTIR) spectral data of blank NPs and mirin-loaded PLGA NP formulation were obtained from lyophilized solid powders (Labconco Freeze Dry System,–48°C, 133 × 10^–3^ mbar) (58). FTIR spectra of samples were obtained between 4000 and 650 per cm on the Universal ATR sampling Accessory plate using a Spectrum 100 FTIR spectrophotometer.

### Tissue distribution of NPs following IP injection.

Athymic male nude mice, 8 weeks old (Jackson Laboratory), were used, and the experiments were conducted in accordance with the IACUC at University of Tennessee Health Science Center (UTHSC). For this experiment, each of the 3 mice were injected into the IP space with PLGA-NPs containing ICG dye (50 μg). After 24 hours, mice were anesthetized with isoflurane and imaged using with a coupled device camera in IVIS XRMS Imaging System (Caliper Life Sciences) (55, 56). To confirm the fluorescence intensity from each organ, the mice were euthanized to harvest various organs for arrangement on a Petri dish. Both in vivo and ex vivo imaging were performed to detect fluorescence levels emitted by the PLGA-NP-ICG at an excitation filter of 690 nm for 5000 ms. The data were presented in photons per second per square centimeter per steradian (p/s/cm^2^/sr).

### Histopathology.

Paraffin-embedded sections (4 μm) were deparaffinized in xylene and rehydrated in increasing ethanol percentages to prepare for staining with hematoxylin and eosin. The kidney sections were deidentified and scored by a blinded nephrologist with expertise in rodent models of AKI to assess tubular injury. The criteria for damaged tubules included the identification of flattened epithelia, tubular dilation, and cast formation as previously described in our lab (23, 25, 26, 59–61). The injured tubules were calculated as a percentage of the total number of tubules counted in 3–5 different sections from every animal in each group.

### Immunofluorescent histochemistry in kidney sections.

Immunohistochemistry was performed on deparaffinized kidney sections (4 μm). Antigen retrieval was performed by heating for 45–50 minutes, and slides were washed in peroxidase blocking solution for 15 minutes. For sections stained with 8-OH-dG, RNase A (Invitrogen, Thermo Fisher Scientific) (20 μg/mL) was added for 60 minutes at 37°C. The tissue sections were washed in 1× TBS with 0.08% Triton X-100 for 10 minutes and blocked in 2.5% normal donkey serum for 60 minutes. Monoclonal GFP antibody (B-2; 1:100-1:200; Santa Cruz Biotechnology catalog sc-9996) or 8-OHdG (1:20 dilution; catalog ab48508; Abcam), and polyclonal Ki67 (catalog 15580, 1:200; Abcam), were incubated on the tissues overnight at 4°C. The tissues were washed 3 times in 1× TBS and the secondary donkey anti–mouse IgG conjugated with Alexa Fluor 555 (1:250 dilution; catalog A-31570; Invitrogen, Thermo Fisher Scientific). To determine specific segments of the nephron, biotinylated PVA-E (1:500 dilution; proximal tubules; Vector Laboratories) or DBA (1:500; collecting ducts) lectin was incubated onto the sections for 30 minutes at 25°C, then with streptavidin-linked IgG with Alexa Fluor 488 (1:500 dilution; Invitrogen, Thermo Fisher Scientific, catalog S11223) for 30 minutes. The sections were mounted with DAPI and imaged on the EVOS light microscope at 20× to 40× original magnification.

### Immunohistochemistry using DAB as the substrate.

Immunohistochemistry was performed on deparaffinized kidney sections (4 μm) using a slightly modified protocol from that previously described (26, 61, 62). In brief, antigen retrieval was performed by heating for 30–50 minutes, incubating in peroxidase blocking solution to minimize endogenous peroxidase activity, and tissue permeabilizing in 1× Tris buffer saline containing 0.08% Triton X-100. The following primary monoclonal mouse antibody against PCNA (catalog 2586; 1:250; Cell Signaling Technology), and primary polyclonal rabbit antibodies against phospho-H2AX (Ser139) (catalog 9718; 1:250, Cell Signaling Technology), phospho–histone H3 (Ser10; catalog 9701; 1:250; Cell Signaling Technology), cleaved caspase-3 (catalog 9661, 1:200; Cell Signaling Technology), and Ki67 (catalog 15580, 1:200; Abcam) were used on the kidney sections. Secondary goat anti–rabbit IgG (Invitrogen, Thermo Fisher Scientific; 1:250) and goat anti–mouse IgG conjugated to horseradish peroxidase (1:250; Cell Signaling Technology catalog 7076) were used and detected using DAB (MilliporeSigma). To determine the localization of GFP to the proximal tubules, PVA-E (Vector Laboratories B-1125) lectin conjugated to biotin was used (1:250). Sections were counterstained with hematoxylin. Pictures were taken with an EVOS light microscope at 20× to 40× original magnification. The percentage of positive nuclei for the target proteins was calculated as a total number of nuclei.

### Western blot analysis.

Protein lysates were isolated from the harvested mouse organs by homogenization in 1× RIPA buffer containing protease and phosphatase inhibitors (Thermo Fisher Scientific) followed by differential centrifugation as previously described (26, 61, 62). Western blot analysis was performed using a 4%–12% SDS-PAGE gradient gel, then transferred to either PVDF or nitrocellular membranes, and bands were detected by chemiluminescence (GE, now Cytiva) using Bio-Rad ChemiDoc MP imaging system. Antibodies used in this protocol were as follows: GFP (catalog sc-9996; Santa Cruz Biotechnology), FLAG (catalog A8592-1MG; MilliporeSigma), Mad2L1 (catalog 4636, 1:1000; Cell Signaling Technology), p31^comet^ (Abcam catalog 150363), TRIP13 (Abcam catalog ab128153), p-p53 (Ser15) (Cell Signaling Technology catalog 9286), and cyclin B1 (Cell Signaling Technology catalog 4135). As a loading control, GAPDH (1:1500; Cell Signaling Technology) was used. All the antibodies were tested using proteins transferred onto PVDF membranes except for cyclin B1 (nitrocellulose).

### Quantitative reverse transcription PCR analysis of gene expression.

Total RNA was extracted using an mRNA isolation kit (Invitrogen, Thermo Fisher Scientific), and cDNA was synthesized using SuperScript III cDNA synthesis kit. Using 1 μg of each RNA, quantitative PCR analyses were carried out in duplicate samples using TaqMan probes, the TaqMan Universal PCR Master Mix (Applied Biosystems, Thermo Fisher Scientific), and a Step One Plus Real-Time PCR System (Applied Biosystems, Thermo Fisher Scientific). The primer set targeting 18S RNA and DNA-PKcs (Mn01342966_g1) was tested on our samples. Relative gene expression quantification was calculated according to the comparative threshold cycle method using 18S rRNA as the endogenous control (63). As a negative control, no template conditions were performed for each primer set to ensure the absence of any spurious DNA amplification by the primer combinations.

### Statistics.

All data are shown as mean ± SEM. The data were tested for normality with the Kolmogorov-Smirnov test using GraphPad Prism 6.0. Normally distributed data were tested with 1-way ANOVA, and subsequent post hoc analysis was performed by Tukey’s multiple-comparison test. Non-normally distributed data were tested with the Kruskal-Wallis test for significance among multiple groups followed by Dunn’s multiple-comparison test. *P* ≤ 0.05 was considered significant.

### Study approval.

Deidentified human kidney sections previously scored as normal or AKI were obtained from the University of Alabama at Birmingham using IRB approval X130124002. All animal protocols (AUA 17-032, 18-097, and 19-0102) were approved by the UTHSC IACUC prior to the start of any of the experiments.

## Author contributions

FP and MMY conceived the idea and designed the study. TH, PKBN, BMM, PC, KRR, and FP conducted the experiments. All the authors contributed to the data analysis and interpretation of the results that were used in the final version of the submitted manuscript.

## Supplementary Material

Supplemental data

## Figures and Tables

**Table 1:**
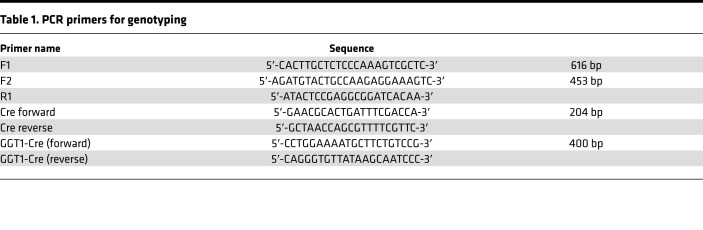
PCR primers for genotyping

## Version 1 11/22/2021

Electronic publication
