# Supplementary material for: DNA damage is overcome by TRIP13 overexpression during cisplatin nephrotoxicity
Source: JCI Insight. 2021 Nov 22;6(22):e139092. doi: 10.1172/jci.insight.139092 (PMC8663775; doi:10.1172/jci.insight.139092)
Supplement: Supplemental data [file jciinsight-6-139092-s008.pdf]

Supplemental Figures

Supplemental Figure 1

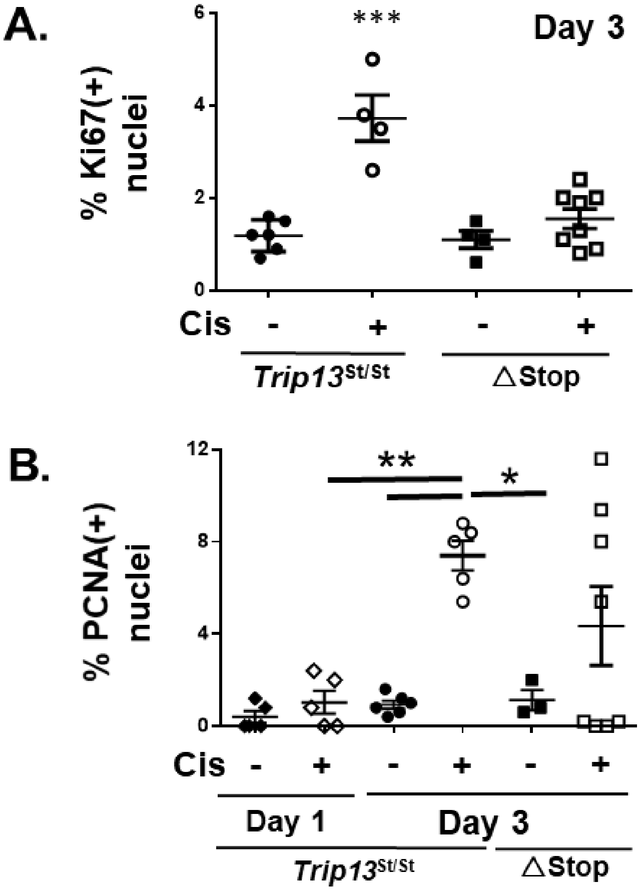

Supplemental Figure 1. TRIP13 did not increase proliferation in normal tubular epithelial cells. Representative sections were stained for Ki67 (A) and PCNA (B) using kidney tissue from *Trip13*$^{Stop/Stop}$ and *Trip13*$^{\triangle Stop}$ female mice treated with either vehicle (V) or cisplatin (C; 15 mg/kg IP) and euthanized after either 1 (PCNA) or 3 days (Ki67 and PCNA) following treatment. Cisplatin treatment significantly increased ($P<0.001$) the percentage of Ki67 nuclei by ~3-fold to 3.7 ± 0.5% (n=4) from 1.2 ± 0.1% (n=6) in vehicle-treated *Trip13*$^{Stop/Stop}$ mouse kidneys. The percentage of Ki67-positive nuclei did not change in the *Trip13*$^{\triangle Stop}$ mouse kidneys regardless of vehicle (1.1 ± 0.2%; n=4) or cisplatin-treatment (1.6 ± 0.2%; n=8), respectively, compared to the vehicle-treated *Trip13*$^{Stop/Stop}$ mouse kidneys. (B) A similar pattern of proliferation was observed with PCNA. Cisplatin treatment modestly increased the proliferative state of the tubular epithelial cells after 24 hours (0.9 ± 1.1%; n=5) compared to the vehicle-treated mouse *Trip13*$^{Stop/Stop}$ kidneys (0.0%; n=5). At 72 hours, there was ~8-fold increase ($P<0.01$) in the PCNA-positive tubular epithelial cells treated with cisplatin (7.4 ± 0.6%; n=6) compared to vehicle-

treated *Trip13*$^{Stop/Stop}$ kidneys (0.9 ± 0.3%; n=6). In the constitutive TRIP13 over-expressing *Trip13*$^{\triangle Stop}$ kidneys, cisplatin tended to increase PCNA-positive cells to 4.4 ± 1.7% (n=8) compared to vehicle treatment (1.8 ± 0.7%; n=4), but the PCNA-positive cells were highly variable. No significant difference was observed between the cisplatin-treated *Trip13*$^{Stop/Stop}$ versus *Trip13*$^{\triangle Stop}$ mouse kidneys. Animal numbers are shown in the graph. *P<0.05; **P<0.01 significant difference between the indicated groups using one-way ANOVA with Tukey's post-hoc analysis. △Stop = *Trip13*$^{\triangle Stop}$ mice; *Trip13*$^{St/St}$ = *Trip13*$^{Stop/Stop}$ mice.

Supplemental Figure 2

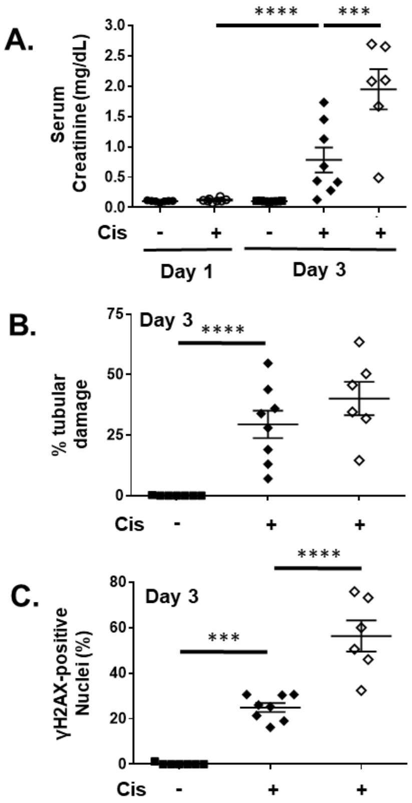

Supplemental Figure 2. Comparative effects of cisplatin between male versus female mice. Cisplatin (15 mg/kg IP) was injected with the same solution in the male (●,○,■,◆) mice as used in the female (◇) mice shown in Figures 1-4. Female blood and tissue values are re-graphed from Figures 2 and 3. Blood and kidney tissues were harvested to measure markers of renal dysfunction. (A) Serum creatinine was measured on days 1 and 3 from male homozygous floxed stop (Trip13$^{Stop/Stop}$) mice after treatment with a single injection of either vehicle (-Cis; 20% captisol) or cisplatin (+Cis; 15 mg/kg IP). A slow increase in serum creatinine was measured in the blood, and there was a significantly lower level of creatinine (P<0.001) in the male versus female mouse sera at day 3. (B) Tubular epithelial cell damage was scored as a percentage of total tubules counted by the same blinded nephropathologist. There was a tendency for the tubular injury score to be lower by ~25% in the male versus female kidneys, but no significance between male and female scores was determined using statistical tests. (C) DNA damage as determined by γH2AX-positive nuclei was quantitatively assessed in the kidney sections. Male kidney sections (24.9 ± 1.9%; n=8) were significantly lower by ~55% than female kidney sections (56.3 ± 7.5%; n=6) treated with cisplatin at day 3. ***P<0.001; ****P<0.0001 significant difference between the indicated groups using one-way ANOVA with Tukey's post-hoc analysis.

Supplemental Figure 3

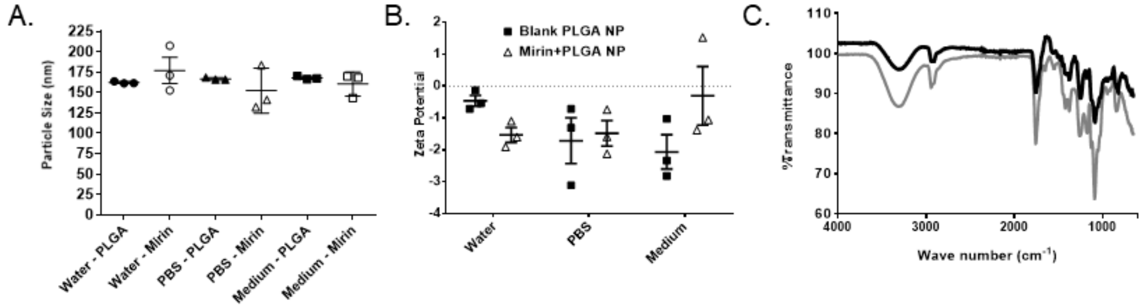

Supplemental Figure 3. Physical characterization of PLGA-mirin nanoparticles. PLGA nanoparticles (NP) were designed to encapsulate the hydrophobic drug, mirin, inside the core of the nanoparticle suspension. PLL and Pluronic F-68 were added to ensure the stability of the nanosuspension. The following assays were performed to confirm the properties of the NP using our formulation. A) Dynamic light scattering (DLS) particle size analysis of PLGA and PLGA-mirin NPs were acquired by Zetasizer (Nano ZS, Malvern Instruments, Malvern, UK) in water, PBS and cell culture medium, respectively. From the DLS data, the encapsulation of mirin inside the core of the nanoparticles (mirin loaded PLGA NPs shown as open symbols) showed no significant difference in particle size or distribution pattern compared to the PLGA NPs (without any drug shown as solid symbols). B) Zeta potential measurement of PLGA and PLGA-mirin NPs was obtained by Zetasizer (Nano ZS, Malvern Instruments, Malvern, UK). Each of the NP formulations exhibited a negative zeta potential in all the mediums suggesting the stability of formulation with minimal to no aggregation during storage. C) Fourier-transform Infrared (FTIR) spectral analysis of PLGA and PLGA-mirin NPs were acquired on a Universal Attenuated Total Reflectance (UATR) accessory plate by spectrophotometry between 4000 and 650 cm^{-1}
(Spectrum 100 FTIR spectrophotometer; Waltham, MA). FTIR spectra revealed the presence of a broad ranged peak between 3600 and 3000 cm^{-1} confirming presence of hydroxyl groups and the glycosidic C–O–C stretching at 1000 cm^{-1} that could be attributed to the Pluronic F-68 polymer (1). Specific peaks for mirin was not detected in by FTIR due to miscibility of drug in the PLGA NPs core and confirming that the drug was encapsulated within the nanoparticles. Black bars/lines = control PLGA NP; grey bars/lines = PLGA-mirin NP.

1. Jain TK, et al. Magnetic resonance imaging of multifunctional pluronic stabilized iron-oxide nanoparticles in tumor-bearing mice. *Biomaterials*. 2009;30(35):6748–6756.

Supplemental Figure 4

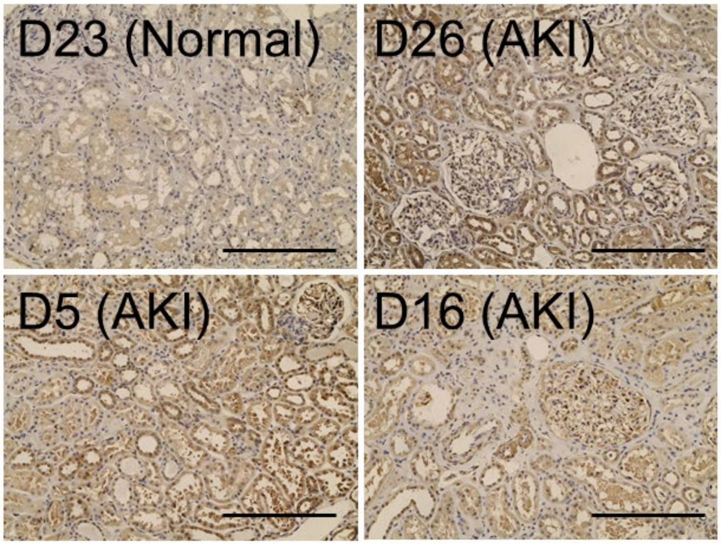

Supplement Figure 4. TRIP13 is expressed in normal and AKI human kidneys. Human kidney sections were obtained from the O'Brien Center clinical core at the University of Alabama in Birmingham. Research authorization was provided by the donor family and was performed under UAB IRB approval (X130124002). Kidney biopsies were obtained using an 18-gauge needle immediately prior to organ retrieval and at the start of cold preservation. The tissue was placed in formalin and embedded in paraffin in standard fashion for tissue sectioning. Human kidneys were scored as (A) normal or (B-D) acute kidney injury (AKI) by an expert nephropathologist. Immunohistochemistry was performed using TRIP13 primary antibody (Cell Signaling cat #ab64964) at 1:200 dilution overnight at 4°C. Goat anti-rabbit IgG conjugated with HRP (1:500 dilution) was added for 90 minutes at room temperature, and after several washes, the TRIP13 protein was detected using DAB precipitation. All slides were counterstained with hematoxylin and imaged using EVOS light microscopy. TRIP13 staining was observed to be relatively ubiquitous through the kidney section, particularly in the renal tubules. Sporadic staining was detected in a few glomerular cells.
